# In the interests of time: improving HIV allocative efficiency modelling via optimal time-varying allocations

**DOI:** 10.7448/IAS.19.1.20627

**Published:** 2016-02-23

**Authors:** Andrew J Shattock, Cliff C Kerr, Robyn M Stuart, Emiko Masaki, Nicole Fraser, Clemens Benedikt, Marelize Gorgens, David P Wilson, Richard T Gray

**Affiliations:** 1 The Kirby Institute, University of New South Wales, Sydney, Australia; 2 The Burnet Institute, Melbourne, Australia; 3 School of Physics, University of Sydney, Sydney, Australia; 4 Department of Mathematical Sciences, University of Copenhagen, Copenhagen, Denmark; 5 The World Bank Group, Washington DC, USA

**Keywords:** allocative efficiency, HIV/AIDS, mathematical modelling, Optima, time-varying optimization, Zambia

## Abstract

**Introduction:**

International investment in the response to HIV and AIDS has plateaued and its future level is uncertain. With many countries committed to ending the epidemic, it is essential to allocate available resources efficiently over different response periods to maximize impact. The objective of this study is to propose a technique to determine the optimal allocation of funds over time across a set of HIV programmes to achieve desirable health outcomes.

**Methods:**

We developed a technique to determine the optimal time-varying allocation of funds (1) when the future annual HIV budget is pre-defined and (2) when the total budget over a period is pre-defined, but the year-on-year budget is to be optimally determined. We use this methodology with Optima, an HIV transmission model that uses non-linear relationships between programme spending and associated programmatic outcomes to quantify the expected epidemiological impact of spending. We apply these methods to data collected from Zambia to determine the optimal distribution of resources to fund the right programmes, for the right people, at the right time.

**Results and discussion:**

Considering realistic implementation and ethical constraints, we estimate that the optimal time-varying redistribution of the 2014 Zambian HIV budget between 2015 and 2025 will lead to a 7.6% (7.3% to 7.8%) decrease in cumulative new HIV infections compared with a baseline scenario where programme allocations remain at 2014 levels. This compares to a 5.1% (4.6% to 5.6%) reduction in new infections using an optimal allocation with constant programme spending that recommends unrealistic programmatic changes. Contrasting priorities for programme funding arise when assessing outcomes for a five-year funding period over 5-, 10- and 20-year time horizons.

**Conclusions:**

Countries increasingly face the need to do more with the resources available. The methodology presented here can aid decision-makers in planning as to when to expand or contract programmes and to which coverage levels to maximize impact.

## Introduction

Despite ambitious targets to end the HIV epidemic, international investment in the global HIV response has plateaued in recent years [1,2]. At the same time, countries are increasingly expected to fund HIV programmes domestically [3,4]. In this context, it is imperative that countries achieve more with available resources by allocating funds as efficiently as possible. Allocative efficiency is the term used to describe the allocation of funding across prevention, treatment, support and other programmes to achieve the greatest possible impact in terms of specific objectives, such as reducing new HIV infections or HIV-related deaths [5]. Allocative efficiency can be quantitatively analyzed via mathematical and economic modelling, using data on epidemiology, programme expenditure and intervention effectiveness (under setting-specific political and implementation constraints). Such analyses can estimate the combination of programmes likely to have greatest impact against defined health objectives and in turn inform resource allocation planning [6,7].

Prioritizing HIV investments towards the most cost-effective programmes (e.g. targeting key affected populations in concentrated epidemics) can lead to substantial epidemiological and economic improvements compared with historical investment approaches [8–11]. Other studies have recently highlighted the importance of geographical prioritization of funds [12]. These modelling studies have generally assumed investments or programme implementation strategies over the analysis period that are constant over time, which may not correspond to the optimal allocation in a given year. Furthermore, these studies have tended to assume changes in funding are instantaneous, whereas in the real world, increases or decreases in funding are necessarily gradual. For example, if an optimized allocation implies doubling of coverage of antiretroviral therapy (ART), this change is likely to occur over several years.

The aim of this study is to advance allocative efficiency methodologies by presenting an approach to calculate the optimal allocation of resources over time across HIV programmes. Our methodology builds upon the previously published Optima model [9]. Optima is a deterministic, population-level HIV-transmission model that has provided allocative efficiency results for over 30 countries [13]. Optima uses non-linear relationships between programme funding and programme outcome indicators to account for initial programme implementation, programme scale-up, economies of scale, and saturation of programme coverage. An optimization algorithm, incorporating pre-defined conditions and constraints, uses these relationships within the epidemic model to determine the optimal distribution of funding across a series of HIV programmes to best meet target objectives. Here, we extend the optimization algorithm to allow allocations to the set of HIV programmes to vary over time within a given total multi-year budget.

Our study aims to assess time-varying optimal resource allocations for both fixed and variable annual budgets and also for various time horizons to assess outcomes. To illustrate the potential real-world benefits of time-varying optimal allocations, we applied our methodology to the HIV epidemic and funding response in Zambia. The HIV epidemic in Zambia is classified as generalized with overall adult prevalence estimated at 13.5% [14]. The primary mode of HIV transmission being heterosexual sex [15]. There has been substantial investment in the Zambian HIV response, with an estimated national HIV expenditure of US$208 million in 2006, rising to US$411 million in 2014 [15,16], coinciding with a 40% reduction in annual new infections between 2005 and 2013 [1]. The vast majority of total national HIV expenditure came from external sources (estimated at 93% in 2012 with PEPFAR and the Global Fund providing the most investment) with the remaining investments from the government and the private sector [15]. Previously, Optima was used to determine an optimal resource allocation for Zambia that was constant over time [15]. Here, we expand the previous analysis using our time-varying allocation methodology.

## Methods

Our methodology can be applied to any HIV-transmission model that incorporates relationships between HIV programme spending and associated risk behaviours or health outcomes. Here, we use the Optima HIV model [9], which uses demographic, behavioural, epidemiological, programmatic and cost data to inform (1) cost-outcome curves that relate programme spending to changes in behavioural and clinical model parameters and (2) a transmission model that is then used to project the impact of changes in programme spending on the HIV epidemic. Optima has previously been used to model the Zambian HIV epidemic [15]. We use the data, model calibration (Supplementary Figures 1 and 2) and cost-outcome curves (Supplementary Figure 3) from this work for our case study.

For spending to be “optimal,” the objectives of the funding need to be defined. Typical objectives of investments in an HIV context are to achieve epidemiological outcomes such as reduced new HIV infections and/or AIDS-related deaths. The optimization period must also be defined. Through the choice of these objectives, an objective function is formed, which can be calculated for any given allocation using relationships between programme spending and outcomes in their targeted populations and the associated projections from the epidemiological model. The optimization algorithm then navigates through the space of possible funding allocations to locate the allocation that minimizes the objective function. The optimization algorithm is run until a minimum is located or further improvements in health outcomes are below a specified threshold (e.g. a 50% reduction in new HIV infections relative to 2014 levels). This process is repeated multiple times using a Monte Carlo initialization to increase the liklihood of locating the global minimum. To perform the optimization, we employ a Bayesian adaptive locally linear stochastic descent algorithm [17].

In allocations that are constant over time, the optimization algorithm works as follows. The allocation to each programme is treated as a parameter in a vector of length *n*, where *n* is the total number of programmes to be optimized. These parameters are constrained such that they must be non-negative and that the sum of all programme allocations is equal to the total budget available at each time point. The optimization algorithm then determines the optimal allocation of spending by evaluating model outputs that result from different possible parameter vectors (i.e. programme allocations). Instead of using a single parameter to represent the funding available to each programme, as described above, the time-varying method uses four parameters. We use a function of the form

$$a(t)=\frac{h\cdot b\cdot e^{-d\cdot t}}{(h-b)\cdot {\text{e}}^{-g\cdot t}+b}$$

to describe how funding to programmes can vary over time. Here, the vectors of initial allocations ***b***=(*b*_1…_*b*_*n*_), the growth rates ***g***=(*g*_1…_*g*_*n*_), the growth thresholds ***h***=(*h*_1…_*h*_*n*_) and the decay rates ***d***=(*d*_1…_*d*_*n*_) are to be optimally determined such that the objective function associated with the allocation ***a***(***t***)=(*a*_1_(*t*) … *a*_n_(*t*)) is minimized. Here, ***t***=(*t*_1…_*t*_*k*_) represents the optimization period of *k* time points, which is mapped onto the closed (normalized) interval [0, 1] and then translated to the actual period of optimization (e.g. the operational budget over the period of a national strategic plan). The initial allocation parameters, ***b***, can range between 0 and the total annual budget available in the first time point of the optimization period, whilst the threshold parameters, ***h***, and the growth and decay rate parameters, ***g*** and ***d***, can take any real values. We illustrate the effect of each of the parameter values in the Supplementary file (Supplementary Figure 5). This function allows allocations to be held constant (Supplementary Figure 6a), “front-loaded” or “rear-loaded” (Supplementary Figure 6b) or initially scaled up/down and then later scaled down/up (Supplementary Figure 6c).

The allocation vector, ***a***(***t***), that arises from the values of the parameter vectors, ***b***, ***g***, ***h*** and ***d***, is normalized such that either of the following occurs:

1. Total programme spending, *T*(*t*_*i*_), equals a pre-defined budget at each time point, *t*_*i*_. The pre-defined budget can be either constant over the optimization period, linearly decreasing or increasing or a step function of likely future annual budgets.
2. Total programme spending across the whole optimization period, $\sum_{i=1}^{k}T(t_{i}),$ is equal to a pre-defined amount, but the total spending at each time point, *T*(*t*_*i*_), is optimally determined. This is achieved by defining a cubic polynomial for total programme spending over time
  $$T(t)=c_{3}t^{3}+c_{2}t^{2}+c_{1}t+c_{0},$$
  where *c*_0_ is equal to the sum of initial allocation parameters *b*_1…_*b*_*n*_, and coefficients *c*_1,_*c*_2_ and *c*_3_ are optimally determined such that (1) the objective function associated with the allocation ***a***(***t***) is minimized and (2) the area under the polynomial is equal to the pre-defined amount of funding available over the optimization period.

Eight direct HIV prevention and treatment programmes were considered in the time-varying optimization process: ART; HIV testing and counselling (HTC); prevention of mother-to-child transmission (PMTCT); voluntary medical male circumcision (VMMC) for adolescent and adult men; condom programmes for men who have sex with men (MSM) and female sex workers (FSW); and prevention programmes for youth and general adults. The estimated national budget for these programmes in 2014 was US$240 million (of the US$411 million total HIV budget). The primary sources of data were the National AIDS Spending Assessment reports, Demographic Health Surveys, UNGASS reports, the 2009 Zambia Sexual Behavior Survey [18–22] and Spectrum and Modes of Transmission input files provided by in-country partners. These data sources are described in detail elsewhere [15].

There are many aspects of planning future funding allocations requiring the consideration of time. First, the expected funding available for all programmes may change over time (e.g. currently many countries are expecting a decline in funding). Second, the achievement of strategic outcomes may be desired in the short, medium or long term. Third, knowing when to feasibly scale programmes up or down is important for implementation purposes. To explore these different aspects, we considered a number of optimization scenarios. For each of these scenarios, we determined the optimal allocation of HIV funding to minimize the cumulative number of new HIV infections using the described time-varying optimization methodology:

1. The optimal allocation between 2015 and 2025 assuming that the 2014 budget is annually available with no predetermined constraints on the amount of funding that could be allocated to each programme.
2. The optimal allocation between 2015 and 2025 assuming that the 2014 budget is annually available with “implementation constraints” where funding to a programme cannot increase or decrease beyond a given proportion per year (we use 30% here) and “ethical constraints” such that anyone who commences either ART or PMTCT cannot cease receiving treatment except by natural attrition.
3. The optimal allocation between 2015 and 2025 where the total spending across the optimization period is equal to that in scenarios 1 and 2, but total annual spending is optimally determined within the restrictions of the implementation and ethical constraints.
4. The optimal allocation of funding over five years but where the cumulative number of new infections is assessed after 5-, 10- and 20-year periods, again with implementation and ethical constraints. Following the period of optimally allocating resources, we assume programmes continue at their levels of coverage attained at the end of the optimization period. Such a scenario may be of particular interest to decision-makers who may only have a short term to impact future health outcomes.

The implementation and ethical constraints in each of the relevant scenarios take effect from the start of 2015. As such, the optimal spending patterns in these scenarios are dependent upon the existing programme allocations. To generate a range of plausible solutions that consider model uncertainty, we repeated the optimization process 40 times by sampling from an ensemble of baseline projections within the uncertainty bounds of the model calibration together with an ensemble of cost-outcome curves within their respective uncertainty bounds. To increase the likelihood of the true optimal allocation being determined, we simulated each scenario a further 40 times. The allocation associated with the greatest epidemiological benefit was then selected as the optimal solution.

## Results and discussion

**Figure 1:**
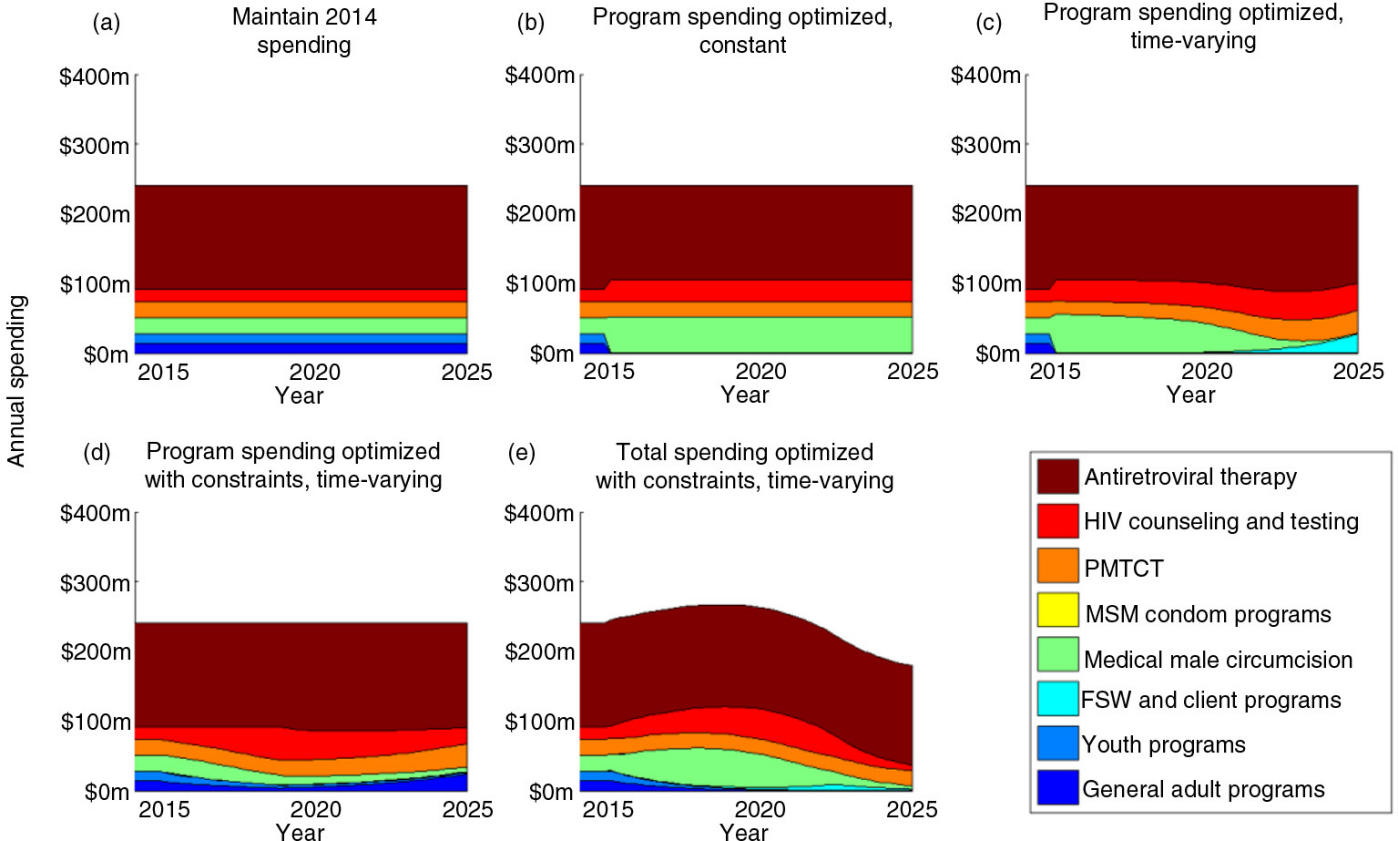
Direct programme spending in Zambia between 2014 and 2025 under different scenarios. The 2014 spending allocation is considered as baseline for the purpose of our scenario comparisons. The plots show optimal redistribution of funds between 2015 and 2025 using (a) no optimization (i.e. maintaining 2014 spending); (b) optimized programme spending that is constant over time; (c) time-varying optimization of programme allocations, with no constraints; (d) time-varying optimization of programme allocations, considering implementation constraints (scale-up/down of programmes capped at 30% per year), and ethical constraints (where ART and PMTCT cannot decrease past 2014 levels); and (e) time-varying optimization of total 2015 to 2025 spending and programme allocations, also considering the same constraints.

**Figure 2:**
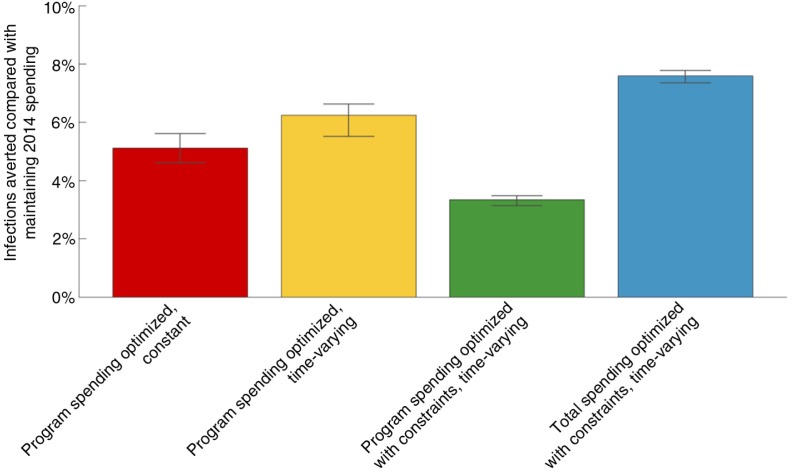
The percentage of infections averted between 2015 and 2025 for each of the scenarios shown in Figure 1 compared with a baseline of maintaining 2014 spending. The uncertainty bars were determined by repeating the optimization process 40 times using an ensemble of 40 projections within the uncertainty bounds of the model calibration with an ensemble of 40 cost-outcome curves within their respective uncertainty bounds (see the Supplementary file for figures illustrating the uncertainty in model calibration and the cost-outcome curves).

Our model of the Zambian HIV epidemic projects an estimated 559,100 (534,800 to 595,000) new HIV infections over 2015 to 2025 if 2014 budget levels and funding allocations across programmes are maintained (Figure 1a). The same level of funding optimized to minimize the cumulative number of new infections but in a non-time-varying manner is estimated to avert 5.1% (4.6% to 5.6%) of these projected new infections (Figure 1b and Figure 2). VMMC and ART programmes are prioritized in this allocation, with funding to PMTCT and HTC remaining at roughly current levels. Allowing the allocation of funds to optimally vary over time further increases the number of infections averted to 6.2% (5.5% to 6.6%) (Figure 1c and Figure 2). In this time-varying optimal allocation, VMMC and ART programmes are initially prioritized, before FSW programmes are scaled up from an initially low level as the VMMC programme is scaled down (since fewer new circumcisions are required to sustain coverage). As with the time-constant optimization, allocations to PMTCT and HTC programmes remain at roughly current levels. Details of these spending allocations and their associated uncertainties are provided in the Supplementary file.

**Figure 3:**
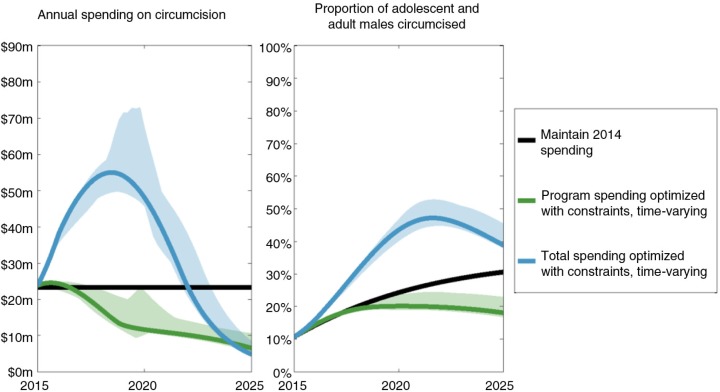
Annual spending on VMMC programmes and the associated change in prevalence of circumcised men. In both optimized scenarios (green and blue curves), implementation constraints (where programme scale-up/down is restricted to a maximum of 30% per year) and ethical constraints (where ART and PMTCT funding cannot be decreased) are applied. In the scenario represented by the green curve, total annual spending is fixed at 2014 levels. In this case, a large initial scale-up of the VMMC programme is not attainable because of the limited availability of unreserved funding and restrictions on programme scale-up/down. Thus, the optimal solution does not prioritize this programme. In the scenario represented by the blue curve, total annual spending is optimally determined such that total spending across the 2015 to 2025 period is the same as in all other scenarios. In this case, total annual spending is initially increased to allow for the initial rapid scale-up of the VMMC programme. Although VMMC spending is later rapidly scaled down, the proportion of circumcised men in this scenario remains considerably higher than in other scenarios.

Constraining programme scale up or down by 30% per year and ensuring ART and PMTCT spending cannot reduce past 2014 levels, the estimated number of new infections averted when allocations are optimally allocated over time is reduced to 3.3% (3.1% to 3.5%) (compared with the baseline of maintaining 2014 spending) (Figure 1d and 2). The rapid initial scale-up of VMMC cannot occur under such constraints (Figure 3), and as such, the programme is not prioritized in the optimal allocation. This negatively influences the projected number of new infections, highlighting the diminishing returns of optimal allocative efficiency when increasingly restrictive constraints are applied. Supplementary Figure 7 shows a finer granularity of implementation constraints as well as the improvement in outcomes with varying levels of funding restrictions. This figure also illustrates the additional gains from optimal time-varying allocations over optimal constant solutions, particularly as constraints are introduced.

By allowing the annual budget to be optimally determined between 2015 and 2025 whilst fixing the total funding to be equal to constant spending at 2014 levels, we estimate that 7.6% (7.3% to 7.8%) of new infections can be averted whilst also adhering to the realistic implementation and ethical constraints. In this optimal allocation of funds, total spending is initially increased to allow for the rapid scale-up of the VMMC programme (within the bounds of the implementation constraint), whilst general adult and youth programmes are gradually scaled down. Following an initial increase, the total programme spending is scaled back to comply with the overall 2015 to 2015 funding restriction, which is achieved by scaling down HTC and, more noticeably, VMMC programmes after 2020.

**Figure 4:**
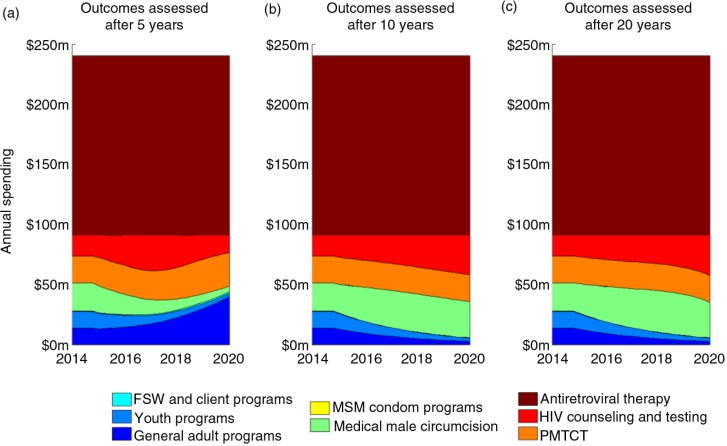
The optimal redistribution of resources when the period of spending is fixed to five years (from 2015 to 2020), and outcomes are assessed after (a) five years, (b) 10 years and (c) 20 years. Under each scenario, implementation constraints (scale-up/down of programmes capped at 30% per year) and ethical constraints (ART and PMTCT cannot decrease past 2014 levels) are observed.

Resource allocations over a period of five years differ according to whether associated epidemiological impacts are assessed after 5-, 10- or 20-year time horizons. For shorter time horizons, the impact of VMMC on HIV outcomes is not realized, and therefore, this programme is prioritized to a lesser degree; instead, there is greater priority for primary prevention for the general population, which has a more immediate effect (Figure 4). However, the allocations remain remarkably similar once the time horizon for impact is 10 or more years (Figure 4). This suggests that considering a 10-year time horizon for programme funding may be sufficient to capture most long-term effects.

Our findings suggest that by considering the varying cost-effectiveness of HIV programmes over time and allocating funds accordingly, reductions in the number of cumulative new infections can be achieved when using data from the generalized epidemic setting of Zambia. We note, however, that the Zambian HIV response is to some degree already allocatively efficient, and therefore, the overall gain of optimizing allocations of funding is modest. The epidemiological gains were most pronounced when the total programme spending was optimally determined rather than held constant over the optimization period, highlighting the potential impact of bringing investments in treatment and prevention programmes forward. Significantly, the epidemiological gains made under the scenario of optimal total programme spending were achieved whilst considering realistic implementation and ethical constraints. In the optimal constant programme spending scenario, these constraints were not considered, and the programme allocations were assumed to be able to scale up instantly. In general, our optimization results here favour large-scale spending on treatment (supported by testing), PMTCT and the front-loaded investment of VMMC programmes. The favoured initial investment of VMMC is likely due to this being a one-time procedure which maintains efficacy in individuals for life, and likely has an additional indirect effect at population level [23]. This finding is consistent with empirical programme evaluations and other modelling studies [24–26].

Our results indicate that fewer health gains occur with increased constraints on annual changes in programme spending (Figure 2; Supplementary Figure 7). We found quickly diminishing returns in the projected epidemiological outcomes from optimal allocations with increasingly tight implementation constraints compared with unconstrained allocations. The implementation constraints discussed in this study were applied to reflect realistic on-the-ground restrictions for the scale-up/down of programmes, although in many settings opportunities may exist to potentially relax these constraints by boosting service delivery capacity through health system strengthening, private sector involvement and performance contracts. One such example has been observed in South Africa, with the implementation of performance-based contracting for general practitioners for VMMC operations [27]. Here, we consider only linear forms of implementation constraints; however, non-linear restrictions on the annual scale-up/down of programmes could also be incorporated into our methodology. The ethical constraints applied in the relevant scenarios are likely to be necessary restrictions for many governments, particularly in the case of ART. Because of both the population-level preventative effect of ART [28–30] and the ethical implications of denying treatment to infected individuals, ART may often be considered an essential programme for which funding cannot be retracted.

Our findings indicate that the short-term optimal allocation of programme funding can vary substantially based on whether associated outcomes are assessed over a short- or long-term period (Figure 4). These findings are significant because of the short-term nature of national governance in many settings, where expenditure may only be controlled over short time horizons. Although decision-makers and other stakeholders generally desire to observe greater outcomes in the shorter term, ultimately the greatest impact should be viewed over longer periods. Indeed, programmes such as infant male circumcision (not considered in this case study) would require particularly long time horizons to assess the epidemiological impacts of investments. Interestingly, our analyses revealed that the optimal allocations when assessing outcomes over 10- and 20-year periods are essentially identical. This may be because of the natural course of HIV infection being around 10 years and that this period covers the duration in which people may be at their greatest risk. Significantly, with the resources available for the national HIV response in Zambia, if the ultimate goal is to minimize disease burden in the long term, then VMMC should be prioritized (along with ART), and our results indicate that the required resources could be made available by gradually scaling down programmes for the general adult and youth populations.

Several limitations of this study exist that could be limiting the epidemiological impact of the optimal allocation solutions. First, our model of the Zambian HIV epidemic considers all individuals to be in a single geographical location. By segregating the modelled population groups by location and redefining the intervention programmes to impact on the relevant individuals, improvements in epidemiological outcomes could be achieved by effectively allocating resources across the geographical regions as well as over time. Second, our study only considers efficiencies through effective resource allocation and does not account for potential technical and implementation efficiency gains. Here, we assumed that the HIV response expenditure outside the eight prevention and treatment programmes was for essential programmes with a fixed cost. In practice, the costs that are not included in the allocative efficiency optimization process – which fund enabling environment, research and other support activities, and sum to around USD$170 million per year in Zambia – would also need to be reviewed in terms of efficiency. Another potential limitation of this study, as with all population-based models, is the granularity with which we define the population groups; an oversimplification of the heterogeneity in risk behaviours within a population can cause inaccuracy in the model findings. Here, we defined the risk groups for our model of the Zambian HIV epidemic based on the availability of population-group-specific demographic and behavioural data and through discussion with partners in Zambia [15].

We deemed the mathematical function used to define the programme allocations over time in this analysis to be the most simplistic function to capture the desired funding dynamics (constant, front-loaded, rear-loaded or initial scale-up/down followed by a later scale-down/up). A limitation of our methodology is that this function may not capture more complex changes in spending patterns. We also considered a simpler function that was capable of capturing front-loaded, rear-loaded and constant allocations over time but not initial scale-up/down followed by a later scale-down/up. This function used two parameters to describe funding dynamics for each programme instead of four parameters used here. Of these two approaches, the four-parameter approach was able to consistently locate an allocation that led to a smaller number of estimated new infections. However, a limitation of the four-parameter approach is that it requires substantially more simulation time to derive optimal solutions. A major factor in this increased simulation time is that the probability of locating the global minimum (i.e. the true optimal solution) is decreased when implementing this more complex approach. Therefore, multiple initial values are randomly chosen, and the optimization algorithm rerun to boost the likelihood that the global minimum is located. It is because of this decreasing likelihood of locating the global minimum with increasingly complex methodologies, coupled with rapidly diminishing returns on epidemiological outcomes from these more complex approaches, that functions with more than four parameters were not considered here. Further analysis of the costs and benefits of different functional forms is provided in the Supplementary file.

## Conclusions

It is necessary for governments to do more with what is available. Optimal allocative efficiency analyses can provide country stakeholders with quantitative evidence to most effectively reallocate resources to achieve – to the greatest extent possible – national goals (or other epidemiological or economic targets) within an estimated future budget. The methodology described here enables optimal allocative efficiency analyses to go a step further by highlighting the potential gains that can be achieved by targeting the right programmes to the right people, at the right time.

## Supplementary Material

In the interests of time: improving HIV allocative efficiency modelling via optimal time-varying allocations
