# Supplementary material for: In the interests of time: improving HIV allocative efficiency modelling via optimal time-varying allocations
Source: J Int AIDS Soc. 2016 Feb 23;19(1):20627. doi: 10.7448/IAS.19.1.20627 (PMC4770825; doi:10.7448/IAS.19.1.20627)
Supplement: In the interests of time: improving HIV allocative efficiency modelling via optimal time-varying allocations [file JIAS-19-20627-s001.pdf]

Supplementary material

This supplementary material complements the main manuscript by providing further technical details of the methodology.

Calibration of transmission model to Zambian HIV epidemic and cost-outcome relationships

Using all available demographic, epidemiological, behavioral, and clinical data [1] we calibrated Optima to the HIV epidemic in Zambia over 2000-2014. This was achieved by varying the input parameters within their uncertainty bounds such that the model projections were within the uncertainty bounds of empirical data on population group prevalence (Figure S1), number of new diagnoses per year, and the number of people on treatment (Figure S2). On inspection, the Optima outputs of HIV prevalence, overall incidence, number of mother-to-child infections, and number of AIDS deaths were found to be similar in magnitude and trend to estimates produced by the Spectrum model. Generally, Optima closely matches the available HIV prevalence and treatment data (we were unable to reconcile the lower prevalence in 10-19 year old males reported in 2001 with the prevalence trends in 10-19 year old females). Based on our calibration we estimated the overall and population level incidence over 2000-2020. See Figures S1 and S2.

Cost-outcome relationships

The relationships between program costs and outcomes, developed in order to conduct resource optimization analysis, are presented in Figure S3 for different program and target populations.

Considering uncertainty in optimal allocations

An uncertainty analysis was undertaken to determine how uncertainties in both the model calibration and the cost-outcome relationships impacted on allocation recommendations. Forty baseline model simulations were sampled from an ensemble of projections within the uncertainty bounds of the model calibration (see Figure S1), as were 40 samples of each of the cost-outcome relations within their respective uncertainty bounds (see Figure S2). The optimization algorithm was re-run (using multiple random initializations) for each of the 40 samples under each of the key scenarios discussed in the manuscript (see Figures 1 and 2). The results of this uncertainty analysis are presented in Figures S4A-D with the subfigures representing optimal allocation determined under each of the key scenarios.

Rapidly diminishing returns from increasingly complex functions

The function used to define program allocations over time in this analysis was selected because it was deemed to be the simplest function able to capture desired funding dynamics (constant, front-loaded, rear-loaded or initial scale-up/down followed by a later scale-down/up). We also considered a simpler function that was capable of capturing front-loaded, rear-loaded and constant allocations over time, but not initial scale-up/down followed by a later scale-down/up. This more simplistic function uses 2 parameters to describe funding dynamics compared with the 4 parameters used by the described function. Of these two approaches, the more complex 4-parameter approach was able to consistently locate an allocation that lead to the least number of estimated new infections. This approach, however,

does require substantially more simulation time to execute. Figure S8 shows the number of function evaluations – that is a single run of the mathematical model - required for the optimization algorithm to attain the optimal allocation in each case. Considering that a single function evaluation takes in the order of 1 second to run, a single processor could attain the optimal constant allocation in around 3 hours, the optimal 2-parameter allocation in around 18 hours, and the optimal 4-paramter allocation in around 55 hours. However, by splitting this load across several processors [2] and/or machines, this process can be significantly shortened. A major factor in this increased simulation time is that the likelihood of locating the global minima (that is, the true optimal solution) is decreased when implementing this more complex approach. As such, multiple optimizations are run – each with different initial conditions - to boost the likelihood that the global minimum is located. An evident limitation of this methodology as a whole is that there is no guarantee of actually locating this global minimum, regardless of how many times the optimization process is repeated. The problem of locating the global minima in a high-dimensional space is not a new problem, however, and has been the subject of intense scientific investigation for some time [3-5]. More generally, it is due to this decreasing likelihood of locating the global minima with increasingly complex methodologies, coupled with rapidly diminishing returns on epidemiological outcomes from these more complex approaches, that overly-complicated functions with many parameters are not considered here, although it would indeed be possible to use any arbitrary function to represent the dynamics of time-varying allocations. We note that our testing has revealed that despite many different initial conditions across the potential solution space, the objective function is not decreased more than when we run this our standard number of times, providing us with confidence that the global minimum is most likely found.

To put this level of computational effort into context, let us consider a non-algorithm approach to determining superior investment allocations. By simply defining 5% increments in coverage from 0% to 100% for each of the eight modeled programs and simulating all possible combinations of program coverage, $21^8 \approx$ 38 billion function evaluations would need to be simulated. To simplistically incorporate time-varying allocations, the number of required function evaluations would be at least 10-100 -fold greater. The most complex of solutions described in this paper required a maximum of 200,000 function evaluations (figure S8). As such, the computational effort to execute the methodology discussed in this paper is at least 2 million times faster than what would be required to attempt optimal allocation analyses without the use of a suitable optimization algorithm such as that employed within the Optima model.

Figure S1: Calibration of Optima to the HIV epidemic in Zambia. Dark grey discs represent available data for HIV prevalence. Lines attached to these discs represent uncertainty bounds. The solid curve is the best fitting simulation, and the shaded region shows the range of the uncertainty simulations.

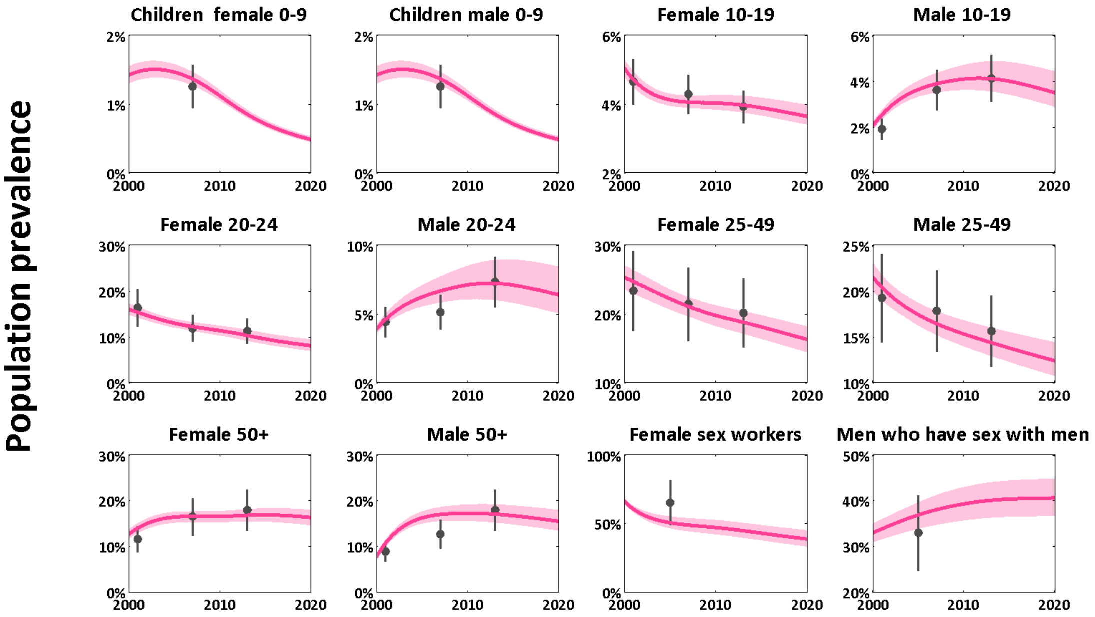

Figure S2: Calibration of model to ART scale-up data in Zambia. Black discs represent available data for the number of people on first and subsequent lines of anti-retroviral treatment. The solid curve is the best fitting simulation and the shaded region represents the range of uncertainty simulations.

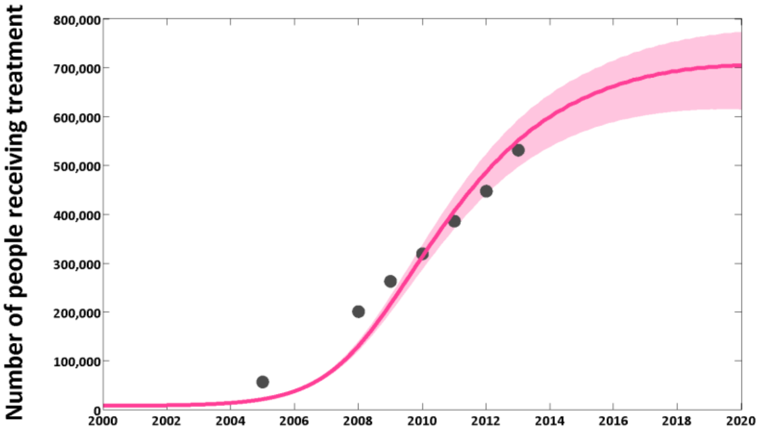

Figure S3: Cost-outcome relationships. Black discs represent available program spending verses program-related outcome data. The solid curve is the best estimate cost-outcome curve, and the shaded region represents the range of uncertainty considered in the each of the cost-outcome relationships.

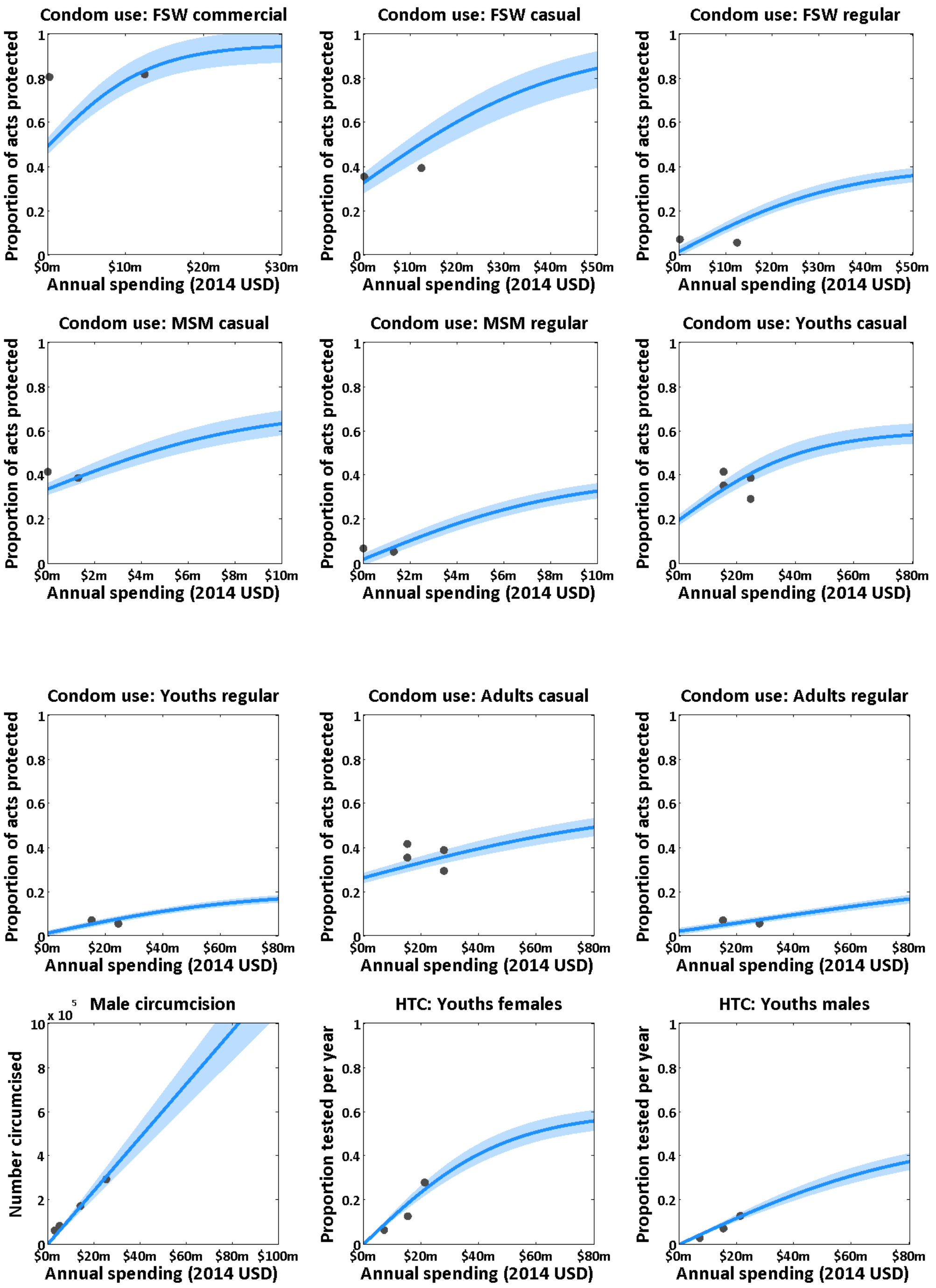

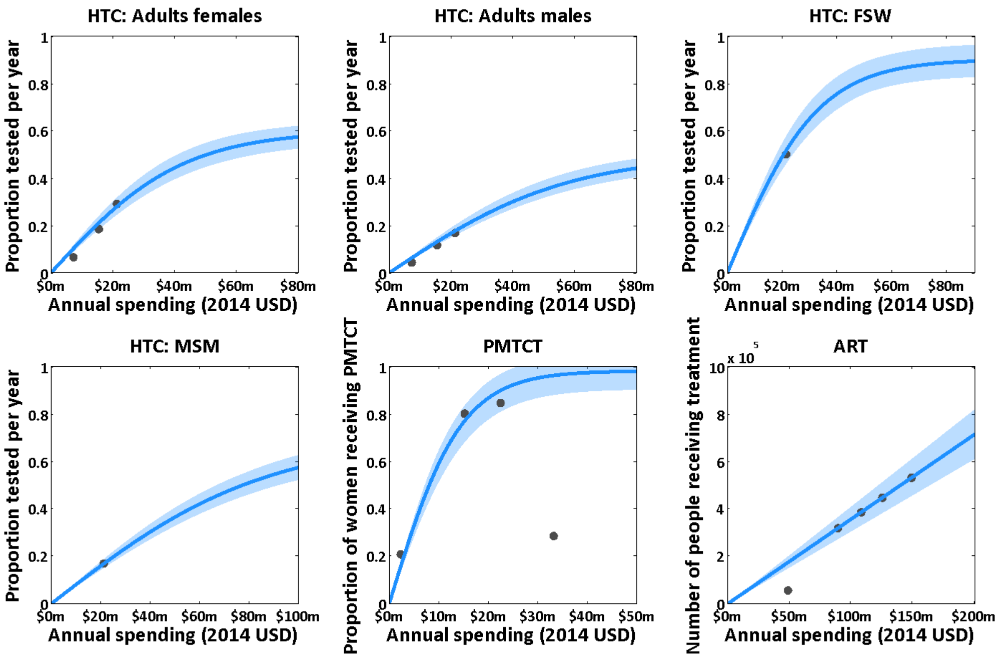
HTC: Adults females
HTC: Adults males
HTC: FSW
HTC: MSM
PMTCT
ART
Proportion tested per year
Proportion of women receiving PMTCT
Number of people receiving treatment
x 10^5
Annual spending (2014 USD)

Figure S4: Impact of uncertainty on optimal allocations under the key scenarios. The bold curves represent the optimal allocation obtained from the analysis of the ‘best-estimate’ model calibration and cost-outcome curves. The shaded regions represent the range of optimal allocations obtained when the 40 samples of model calibration and cost-outcome relations were considered in the optimization process.

Figure S4A: Impact of uncertainty on optimal allocations when using a time-constant optimization approach without constraints on program scale-up/down. In this scenario, total program annual spending is fixed at 2014 levels.

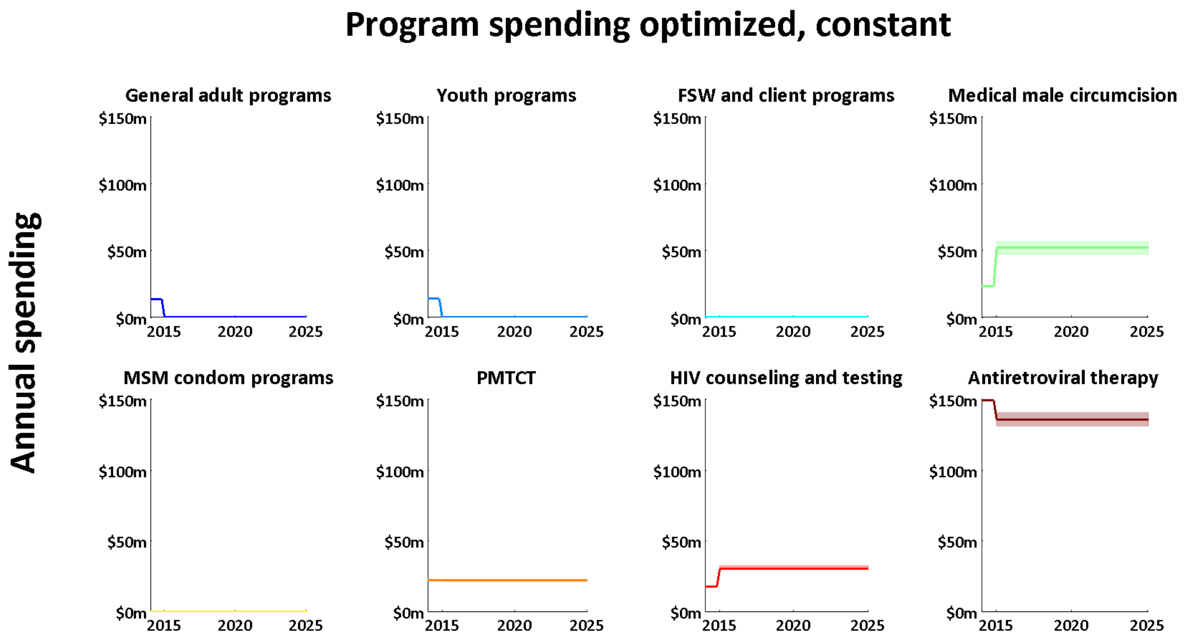

Figure S4B: Impact of uncertainty on optimal allocations when using a time-varying optimization approach without constraints on program scale-up/down. In this scenario, total program annual spending is fixed at 2014 levels.

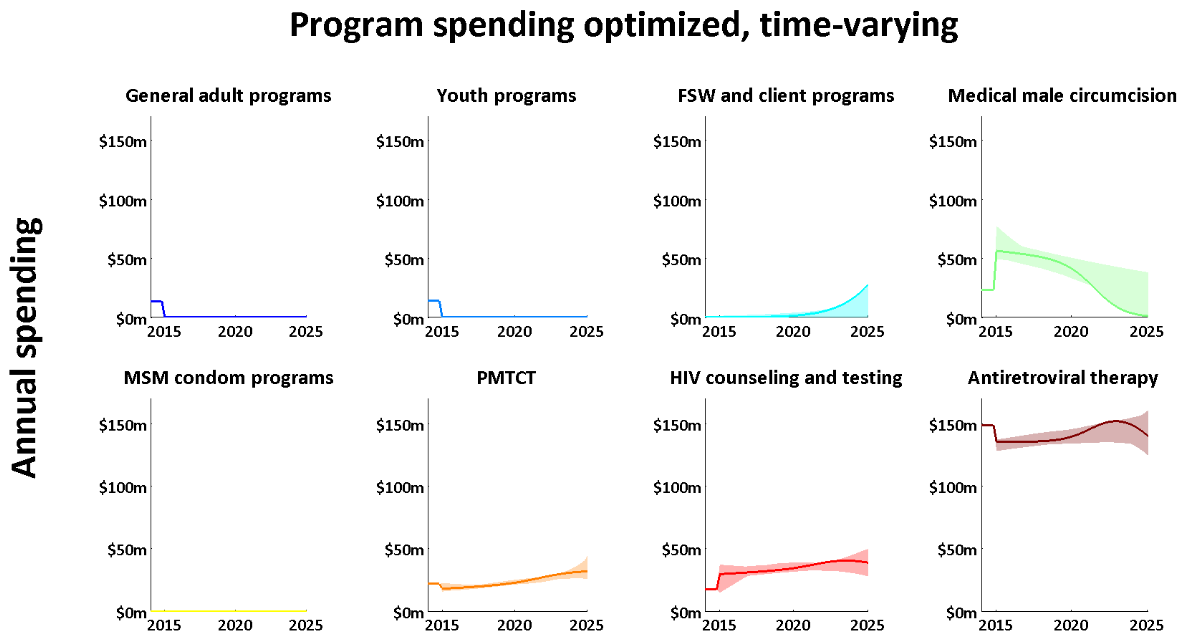

Figure S4C: Impact of uncertainty on optimal allocations when using a time-varying optimization approach with implementation constraints (where funding to a program cannot increase or decrease beyond a 30% per year) and ethical constraints (where anyone who commences either ART or PMTCT cannot cease receiving treatment except by natural attrition). In this scenario, total program annual spending is fixed at 2014 levels.

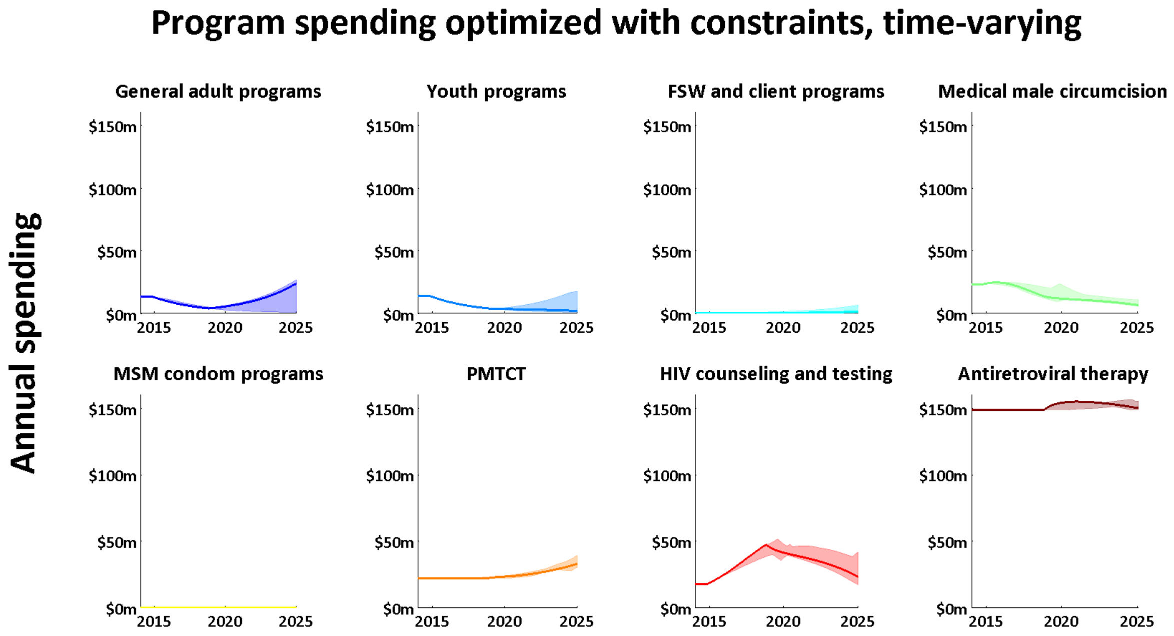

Figure S4D: Impact of uncertainty on optimal allocations when using a time-varying optimization approach with implementation constraints (where funding to a program cannot increase or decrease beyond a 30% per year) and ethical constraints (where anyone who commences either ART or PMTCT cannot cease receiving treatment except by natural attrition). In this scenario, total program is optimally determined such that total program spending over the 2015-2025 period is equal to constant funding at 2014 levels.

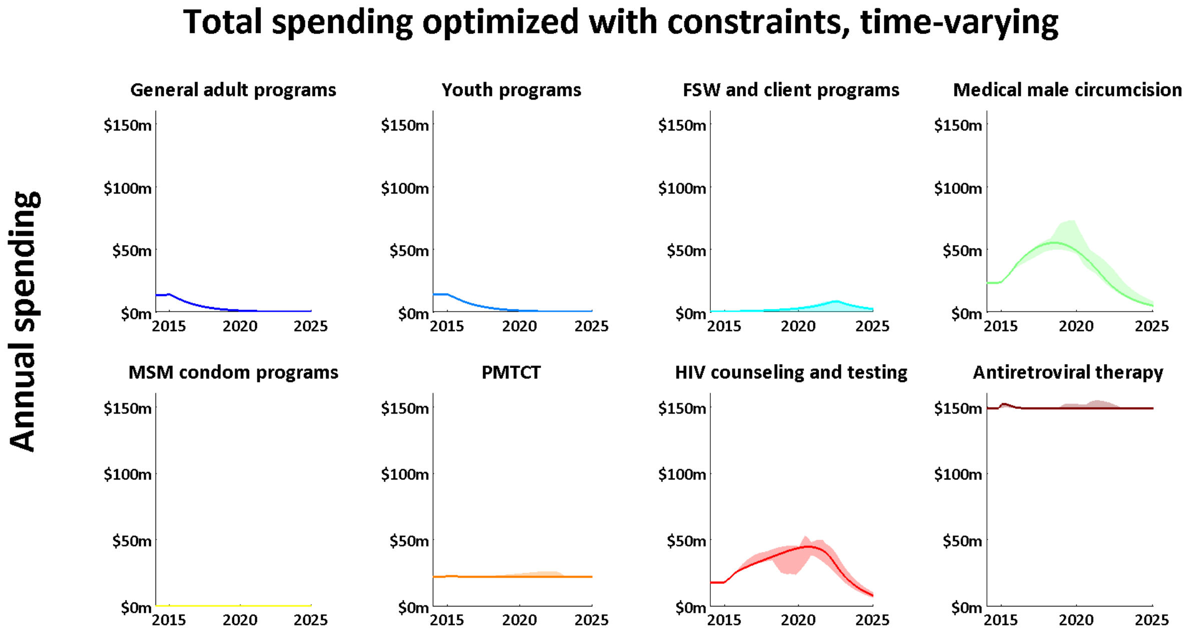

Figure S5: Schematic figure illustrating the effects of each of the function parameters: the initial allocation, b, the growth rate, g, the growth threshold, h, and the decay rate d. In each curve, the initial allocation parameter is set to 30, and the growth threshold parameter is set to 100. In the grey curve, the value of the growth threshold parameter is irrelevant as both the growth and decay rates are set to 0.

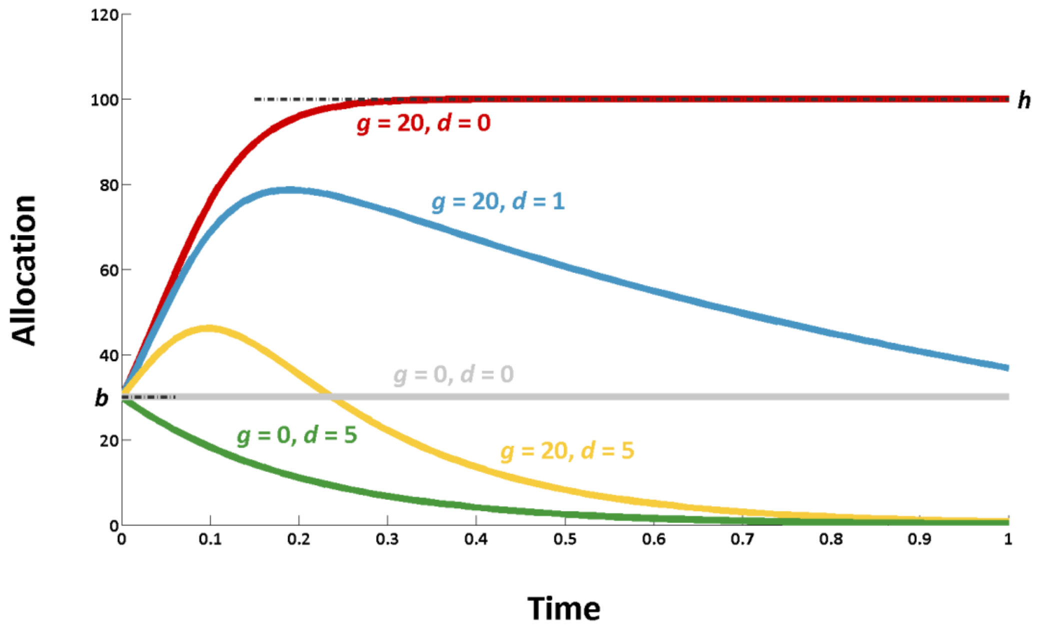

Figure S6: Methodology figure illustrating: A) constant allocations; B) “front-loaded” and “rear-loaded” allocations, and C) allocations that are scaled-up/down followed by a later scale-down/up.

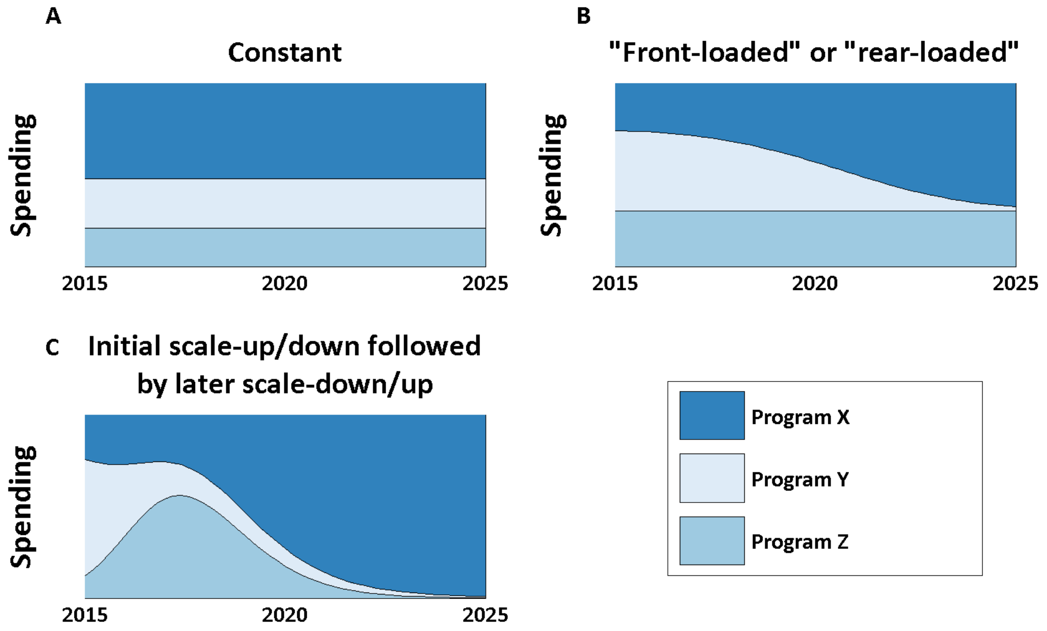

Figure S7: The impact of optimal spending on infections averted between 2015 and 2025 compared to a baseline of maintaining 2014 spending, under varying implementation constraints (that is, the scale-up/down of each program is constrained to be within a percentage of annual spending each year). Each scenario is replicated using a constant allocation methodology, a time-varying methodology using 2-parameter vectors, and the time-varying methodology described in the manuscript (that uses 4-parameter vectors).

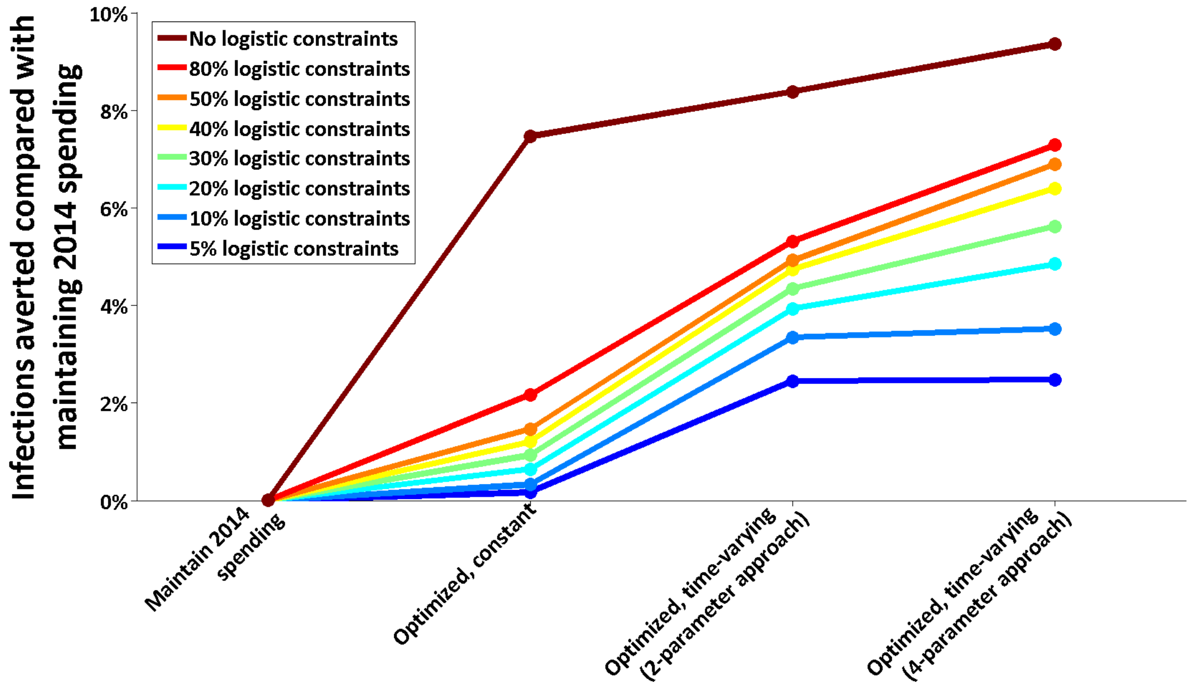

Figure S8: The number of function evaluations required to determine the optimal allocation by implementing the constant spending methodology, the 2-parameter time-varying methodology, and the 4-parameter methodology described in the manuscript.

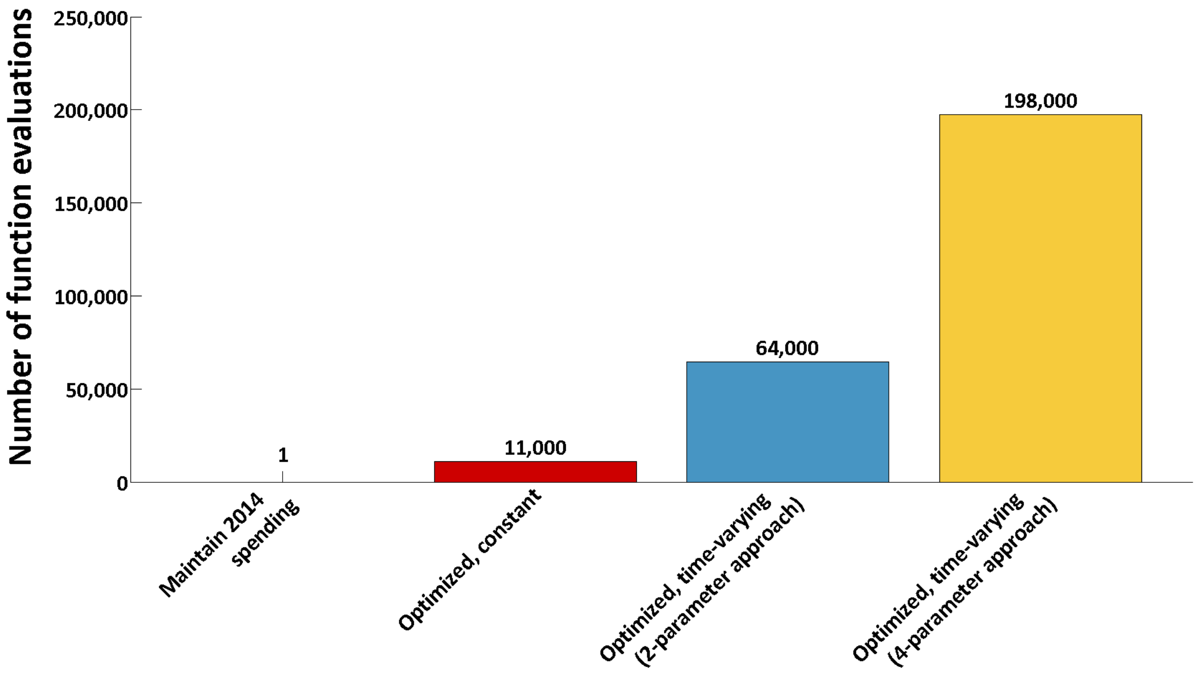

Table S1: Summary of the key scenarios represented in Figures 1 and 2.

Type of optimization	Implementation constraints	Ethical constraints	Total budget assumptions	Optimization time frame	Cumulative new infections over the optimization time frame
Maintain 2014 spending	None	None	Constant total budget	10 years	559,100 [534,800 - 595,000]
Constant allocation optimization	None	None	Constant total budget	10 years	530,600 [506,000 - 566,500]
Time-varying optimization	None	None	Constant total budget	10 years	524,300 [501,200 - 560,600]
Time-varying optimization	30%	ART and PMTCT funding constrained	Constant total budget	10 years	540,500 [517,000 - 575,800]
Time-varying optimization	30%	ART and PMTCT funding constrained	Total budget optimally determined	10 years	516,700 [494,400 – 550,800]

References

1. Masaki E, Fraser N, Haacker M, Obst M, Wootton R, et al. (2015) Zambia's HIV response: Prioritized and strategic allocation of HIV resources for impact and sustainability (findings from the HIV allocative efficiency study).
2. Sharma G, Martin J (2009) MATLAB®: a language for parallel computing. International Journal of Parallel Programming 37: 3-36.
3. Roweis ST, Saul LK (2000) Nonlinear dimensionality reduction by locally linear embedding. Science 290: 2323-2326.
4. Saul LK, Roweis ST (2003) Think globally, fit locally: unsupervised learning of low dimensional manifolds. The Journal of Machine Learning Research 4: 119-155.
5. Storn R, Price K (1997) Differential evolution–a simple and efficient heuristic for global optimization over continuous spaces. Journal of global optimization 11: 341-359.
